# Evaluating the Appropriateness of ENT Emergency Clinic Referrals to Enhance the Quality of Healthcare Provision in the National Health Service (NHS)

**DOI:** 10.7759/cureus.52547

**Published:** 2024-01-19

**Authors:** Abubaker Elamin, Amena Al Saad, Giridharan Wijayasingam, Wai Sum Cho

**Affiliations:** 1 General Surgery, Humanitas University, Milan, ITA; 2 Otolaryngology, Nottingham University Hospitals, Nottingham, GBR; 3 Otolaryngology, United Lincolnshire Hospitals, Lincoln, GBR; 4 General Surgery, Nottingham University Hospitals, Nottingham, GBR

**Keywords:** general practitioner, referral and consultation, junior doctor training, emergency clinic, nhs, otolaryngology training, ear nose throat (ent)

## Abstract

Background

Ear, Nose, and Throat (ENT) services in the National Health Service (NHS) face escalating pressure, exacerbated by the COVID-19 pandemic, resulting in prolonged waiting times and increased referrals. Understanding the factors driving pressure on ENT services is crucial for enhancing patient care and resource allocation.

Methods

A retrospective single-centre cohort study was conducted at Queen's Medical Centre, Nottingham, UK, over five weeks. A total of 156 referrals to the ENT Emergency Clinic (E-Clinic) were analyzed, assessing the appropriateness of referrals and healthcare professionals' involvement in reviewing cases.

Results

The analysis revealed 28 distinct case categories, with certain conditions being predominant in specific reviews (e.g., otitis externa, nasal fractures, epistaxis). Notably, 21.8% of cases were deemed unsuitable or inappropriate for E-Clinic assessment. Strategic restructuring was suggested, distributing cases among healthcare professionals based on expertise and complexity.

Discussion

The findings underscore the need for a refined referral process and appropriate allocation of cases, emphasising the importance of nurse-led reviews for certain conditions and the necessity for senior review in complex cases. Improving the primary-secondary care interface and educating healthcare professionals on appropriate referrals are crucial for refining the system.

Conclusion

Optimising the quality of referrals and allocation of cases within ENT E-Clinics can alleviate workload pressures and enhance patient care. Strategic distribution of cases based on expertise and complexity, alongside refined referral processes, can significantly improve clinic efficiency and patient outcomes in the NHS.

## Introduction

ENT in the National Health Service (NHS)

Within the NHS of the United Kingdom, Ear, Nose, and Throat (ENT) services vary in their delivery across regions due to several influencing factors. These specialized departments cater to a spectrum of medical needs, offering elective and emergency services that encompass inpatient, day case, and outpatient care. The comprehensive range of services within ENT clinics spans adult and pediatric specialties, encompassing emergency care, hearing disorders, neck lumps, fractures, sleep studies, and nurse-led clinics [1]. However, the landscape of ENT services has faced mounting challenges, particularly in the wake of the COVID-19 pandemic, characterized by prolonged waiting periods for treatment and a surge in referrals [2,3]. Notably, the availability and readiness of ENT services within the NHS exhibit variations among different trusts (organizational units within the National Health Services of England and Wales, generally serving either a geographical area or a specialized function), with tertiary centres typically offering more comprehensive facilities compared to district hospitals [1,4].

Factors driving pressure on ENT services

There is an ever-increasing workload on ENT departments every year with around 330,000 admissions and approximately 2.8m outpatient attendances, of which 960,000 involve outpatient procedures [5]. Amidst the multifaceted landscape of ENT services, several factors contribute to the strain experienced by these specialized departments. A significant contributing factor is the level of ENT knowledge among primary care providers. Studies have highlighted the challenges faced by general practitioners (GPs) in making accurate referrals, attributing these difficulties to limitations in pre-graduate and post-graduate ENT education [6].

Emergency presentations are quite high within the ENT specialty and studies have shown that emergency workload constitutes a significant portion of aspects of referrals, admissions, and procedures [7]. Consequently, the ENT Emergency Clinic (E-Clinic) serves as a pivotal healthcare institution offering expedited access to address semi-acute conditions. Its swift intervention plays a crucial role in promptly managing health issues, thereby significantly enhancing patient care. The clinic's commitment to rapid response aligns with its dedication to upholding high clinical standards, ensuring substantial benefits for patients in urgent need of medical attention [8].

Importance of timely intervention

The timely intervention provided by the E-Clinic not only addresses immediate health concerns but also mitigates potential complications, underscoring its steadfast commitment to prioritizing patients' well-being. However, the clinic often experiences a substantial influx of routine cases that may not necessarily require urgent attention. While acknowledging the importance of each patient's concerns, addressing this influx of inappropriate cases poses challenges in maintaining the clinic's accuracy and efficiency [8].

Challenges and the need for balance

This surge in routine cases strains resources unnecessarily, potentially impeding the clinic's ability to allocate resources effectively and provide optimal care to individuals in urgent need. Hence, striking a delicate balance between managing urgent cases and appropriately addressing routine cases becomes imperative to ensure an efficient and effective healthcare delivery system within the clinic [9].

Our primary goal was to scrutinize and assess the cases referred to the clinic, aligning them with clinical priorities. This assessment endeavours to reinforce the clinic's commitment to maintaining the utmost standards of safety, quality, and effectiveness in clinical practices. By meticulously examining each referral based on its clinical urgency, the study aims to streamline the process, ensuring judicious allocation of resources and the provision of the most suitable level of care to each patient. Our research was prompted by the escalating pressure experienced by ENT services, leading us to evaluate the quality of referrals directed specifically to the ENT emergency clinic. Our main aim was to assess the suitability of these referrals.

## Materials and methods

This retrospective single-centre cohort study aimed to assess the appropriateness of referrals made to the ENT E-Clinic at Queen's Medical Centre, a tertiary healthcare facility in Nottingham, UK. The study was conducted over a duration of five weeks, specifically from October to November 2023, to comprehensively evaluate the nature and suitability of referrals directed to the E-Clinic. A meticulous examination of medical records within the ENT department was undertaken to retrieve and analyze referral and admission details. These records encompassed a wide spectrum of cases referred to the E-Clinic during the specified time frame. The total number of referrals made during this period was recorded and constituted the dataset for the study, comprising 156 cases. Each referral was subjected to a detailed investigation and assessment to ascertain its nature, clinical characteristics, and urgency. A nuanced approach was employed to categorize and sub-categorize these cases based on the specific medical condition presented. This classification process facilitated the identification of distinct case categories and allowed for a granular understanding of the diverse array of conditions referred to the E-Clinic.

The study evaluated the level of expertise and involvement of different healthcare professionals in the review process of these referrals within the E-Clinic setting. Specific attention was paid to discerning the roles played by specialist registrars, Senior House Officers (SHOs), and ENT nurse specialists in assessing and managing the referred cases. This analysis aimed to identify patterns in the distribution of cases among healthcare professionals and to determine the suitability of different professional levels for managing specific medical conditions. Descriptive statistics were employed to quantify the distribution of referrals across various case categories and the involvement of different healthcare professionals. Frequencies and percentages were calculated to elucidate the prevalence of specific conditions and the proportion of cases managed by different professionals within the E-Clinic.

## Results

This investigation encompassed a comprehensive analysis of a substantial dataset, comprising a total of 156 referrals that were meticulously scrutinized and evaluated. These referrals spanned a diverse spectrum, encapsulating 28 distinct case categories, reflecting the multifaceted nature of medical conditions referred to the clinic under study.

A detailed examination into the classification and sub-categorisation of these cases involved a nuanced assessment to determine the healthcare professional responsible for the review process. This categorization highlighted the involvement of various medical professionals, including specialist registrars, SHOs, and ENT nurse specialists, each playing a distinct role in evaluating the referred cases.

**Figure 1:**
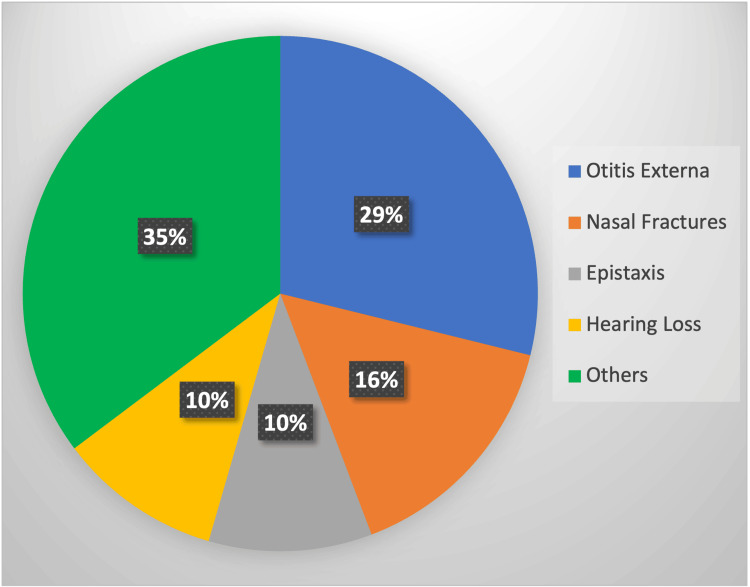
The data have been represented as % E-Clinic Referral Categories

Of particular interest was the identification that a substantial majority, accounting for 64.74% of the total cases, stemmed from only four specific referral categories: otitis externa, nasal fractures, epistaxis, and hearing loss (Figure 1). Notably, within these categories, the examination revealed an intriguing pattern. While the majority of these cases were managed within SHO-led clinics, a distinct outlier was the category of epistaxis (16 cases), predominantly reviewed by ENT Specialty Nurses, comprising approximately 10.25% of the total referrals (Figure 1). 

Other prevalent conditions within this review included foreign body (10 cases), otitis media (five cases), vertigo (three cases), micro-suctioning (three cases), furuncle (three cases), suture removal (four cases), popewick removal (four cases), and splint removal (three cases). There were several singular instances of less common conditions such as ear cysts, abscess of the ear, lump behind the ear, throat obstruction sensation, ear pain, and BAHA (Bone Anchored Hearing Aids) infection, neck lump, and cochlear implants.

However, certain conditions were deemed unsuitable for E-Clinic review due to their acute nature, including pinna haematoma (five cases), pinna cellulitis (one case), perichondritis (one case), scope to assess vocal cord palsy post endarterectomy (one case), and facial droop (one case). Further conditions were considered inappropriate for E-Clinic review consisting of a blocked eye case (one case) and a lytic lesion external auditory canal as a possibility of temporal bone squamous cell carcinoma or necrotizing otitis externa (one case).

## Discussion

The findings gleaned from the comprehensive analysis of referrals within diverse healthcare professional reviews present insightful implications for optimizing the allocation of cases within the clinical setting. The distribution of cases among SHOs, senior-led reviews, and nurse-led reviews, as well as those deemed unsuitable or inappropriate for E-Clinic assessment, underscores the need for a strategic restructuring of the referral process.

**Figure 2:**
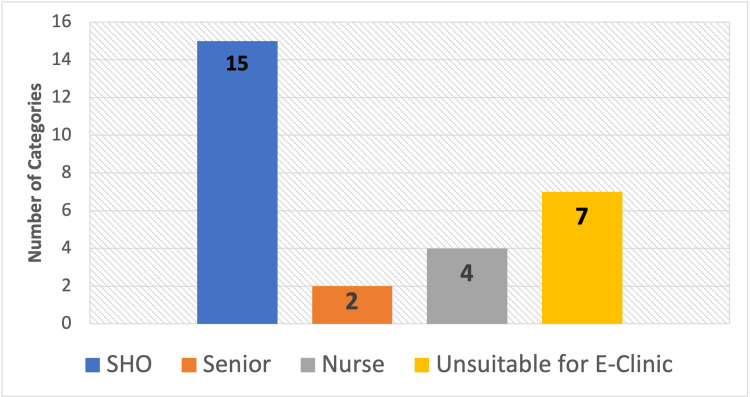
The data have been represented as N Distribution of Referral Categories SHO: Senior House Officer; E-Clinic: Emergency Clinic

It is evident that certain medical conditions tend to recur more frequently within specific reviews. For instance, conditions like otitis externa, nasal fracture, and hearing loss predominated within the SHO-led review, suggesting a pattern where these cases might be more aptly addressed by SHOs. Conversely, the prevalence of epistaxis cases within the nurse-led clinic indicates a specialization that allows nurses to proficiently manage such cases. ENT specialist nurses, not available in every trust, are proving to be capable of reviewing certain case categories which can be utilized to redirect suitable cases. A total of four categories are identified as nurse-led suitable review: epistaxis, suture, popewick, and splint removal (Figure 2). Furthermore, a total of two categories, neck lumps, and cochlear implants, were deemed necessary to be seen by a senior due to the complexity and expertise required in assessing these cases (Figure 2). This recommended distribution within the E-Clinic will ensure safe patient review and alleviate the load of referrals, creating further booking spaces for patients to be seen. 

Moreover, the comprehensive analysis of the referral categories showed a notable fraction of the cases, precisely 7 out of the 28 categories (21.8%), were deemed unsuitable or inappropriate for assessment within the E-Clinic environment under investigation (Figure 2). This discernment underscored the need for a more refined and selective approach in the categorization and referral process, ensuring that only cases suitable for E-Clinic evaluation were directed for assessment within this specialized setting.

This observed distribution prompts consideration for reassigning cases to more specialized professionals. Allocating cases based on their complexity and the expertise required for their assessment could significantly alleviate the burden on SHOs, allowing for a more balanced workload distribution and training sustainability. This notion is further supported by a review of service improvement in healthcare organizations commending leadership interchange among professionals of various levels [10]. It stands to reason that directing cases to the appropriate healthcare professionals, such as senior specialist registrars or specialist nurses, could optimise the clinical workflow, potentially reducing referral backlogs and facilitating increased patient bookings [9].

Furthermore, addressing inappropriate referrals is crucial in ensuring efficient patient care. The identification of conditions unsuitable for E-Clinic review highlights the need for meticulous scrutiny in the referral process to prevent overburdening the clinic with cases that fall outside its scope. These inappropriate referrals not only contribute to backlog but also hinder the timely intervention of cases by the suitable specialty, potentially impacting patient outcomes.

To streamline the referral process, it is imperative to develop a refined system that categorizes cases based on their complexity and appropriateness for specific healthcare professional reviews. A study has shown the implementation of a nurse-led triage facility for easy access has proven to reduce average waiting time by 70% and inappropriate referrals by 50% [8]. Such strategic restructuring can foster an environment where cases are directed to the most suitable professionals, optimizing clinic efficiency, reducing backlogs, and ensuring timely and appropriate interventions for patients in need. 

Additionally, the standards of the referrals can be further polished by addressing gaps within healthcare professionals’ ENT knowledge in various settings. This can be divided into two concepts: improving the source of sending referrals and improving the source of accepting referrals [11]. With regard to the sending source, primary care is a main source of case influx, hence why it is crucial to optimise the primary-secondary care interface. Survey-based research was carried out on GPs to assess their views regarding ENT training which reflected a significant variability with an overall 75% favouring the need for further training [6]. This portrays inadequate ENT teaching and raises considerations for action points in medical education nationally. 

Furthermore, doctors and other healthcare professionals should be advised to manage ENT presentations based on specific national guidelines. This can be attributed to NICE guidelines for the relevant condition, and only escalate if initial steps fail or there are concerns for deterioration. Not to mention that ENT UK provides up-to-date information, resources, and guidelines about the ENT specialty. In their 2021-2022 annual report, ENT UK released the ‘Adult acute severe sore throat management guidelines for emergency department doctors’, which was created for non-ENT doctors whether in primary or emergency care to improve the quality of care [12]. Alongside these guidelines, it is also important to acknowledge and differentiate the cases that require urgent assessment and treatment in Accident and Emergency (A&E) rather than E-Clinic, which is crucial for patient safety.

On the other hand, the doctors accepting the referrals should be further educated on which cases are deemed fit to be seen in an E-Clinic and those that should be directed either back to primary care, A&E, or another specialty if applicable. As junior doctors are mainly the ones to handle incoming referrals, it is noteworthy that background knowledge and experience can widely differ, hence, a baseline of the job expectation and requirements per trust should be outlined. Where this is usually done during induction, it is essential to provide appropriate training for out-of-hours cover and raise awareness on the responsibilities and expected and accepted referrals within a particular trust. This is seconded by the ENT GIRFT (Getting It Right First Time) Programme which aims to evaluate ENT services nationally and provides recommendations based on statistical evidence [13]. A study has shown a reduction in overbookings and inappropriate referrals, 33.7% and 9.9% respectively, with the involvement of the senior registrar during morning hours and then directed to the junior doctor when taking referrals [14]. Whilst showing significant findings, the capacity to involve senior registrars during on-call day and night shifts varies hugely between trusts. However, such intervention can be considered initially for a short period with new incoming junior doctors with a middle-grade or a senior registrar. This can provide support, guidance, and a weaning approach to the responsibilities of the job.

Despite the significant outcomes noted from our study, it does come with some limitations. First, the study, whilst having a high number of cases with significant results, was carried out over a relatively short period of time. However, it proves that with this high case load, one can draw a very thorough and diverse study if done over the span of months. Secondly, there was no outlook on the exact source of referrals for each case obtained which could have given an in-depth picture of the quality and trace-back for gap improvement. Thirdly, the study did not take into light the level of the junior doctor and experience in ENT when accepting these referrals. This could skew the quality as doctors rotate between specialties and the chance of having a doctor with scarce ENT experience. It will also be worthwhile to investigate for any recurrent attendances to the E-Clinic and consider review by a senior clinician to help curb unnecessary excess bookings [15].

## Conclusions

With the ever-rising workload faced by ENT departments within the NHS, ENT Emergency Clinic service utilisation has been sub-optimised and the assessment of referrals has provided crucial insights into enhancing the quality of healthcare provision. This comprehensive study identified diverse case categories and their respective review distribution by different healthcare professionals, emphasising the need for a more refined and strategic approach to managing referrals and specialised allocation based on expertise and complexity. Implementing a more refined system that categorises cases accurately could optimise clinic efficiency, reduce backlogs, and ensure appropriate interventions for patients.

Strategic restructuring of the referral system and efforts to address gaps in ENT knowledge among healthcare professionals, both in sending and accepting referrals, is imperative. The quality of the referrals can be enhanced by conditioning the trajectory between primary and secondary care and providing comprehensive guidelines for managing ENT presentations. A decrease in the workload and appropriate management can be obtained via an apt distribution of cases on a seniority and experience basis, ranging from nurse specialists to senior registrars. Educating doctors and providing appropriate ENT training can further refine the referral process. Overall, raising awareness of ENT training and utilisation of staff and resources will increase the understanding of the referral system for better healthcare provision nationally. 
